# BILE: A Literature Review Based Novel Clinical Classification and Treatment Algorithm of Iatrogenic Bile Duct Injuries

**DOI:** 10.3390/jcm12113786

**Published:** 2023-05-31

**Authors:** Dimitrios Symeonidis, Konstantinos Tepetes, George Tzovaras, Athina A. Samara, Dimitrios Zacharoulis

**Affiliations:** Department of Surgery, University Hospital of Larisa, Mezourlo, 41221 Larisa, Greece

**Keywords:** iatrogenic bile duct injuries, classification systems, Roux en Y hepaticojejunostomy

## Abstract

Purposes: The management of patients with iatrogenic bile duct injuries (IBDI) is a challenging field, often with dismal medico legal projections. Attempts to classify IBDI have been made repeatedly and the final results were either analytical and extensive but not useful in everyday clinical practice systems, or simple and user friendly but with limited clinical correspondence approaches. The purpose of the present review is to propose a novel, clinical classification system of IBDI by reviewing the relevant literature. Methods: A systematic literature review was conducted by performing bibliographic searches in the available electronic databases, including PubMed, Scopus, and the Cochrane Library. Results: Based on the literature results, we propose a five (5) stage (A, B, C, D and E) classification system for IBDI (BILE Classification). Each stage is correlated with the recommended and most appropriate treatment. Although the proposed classification scheme is clinically oriented, the anatomical correspondence of each IBDI stage has been incorporated as well, using the Strasberg classification. Conclusions: BILE classification represents a novel, simple, and dynamic in nature classification system of IBDI. The proposed classification focuses on the clinical consequences of IBDI and provides an action map that can appropriately guide the treatment plan.

## 1. Introduction

**Figure 1:**
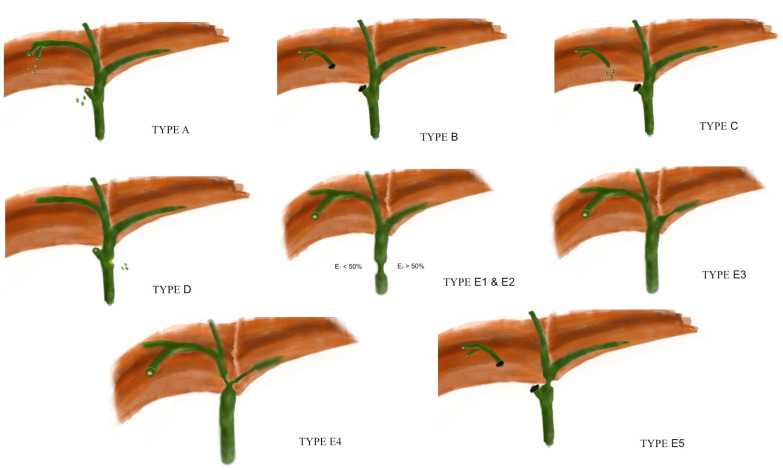
The Strasberg classification of IBDI.

Iatrogenic bile duct injuries (IBDI) represent a challenging complication following gastrointestinal surgery. In general, two major groups of surgical procedures can lead to IBDI. The first group includes procedures performed on the extrahepatic biliary tree, such as open and laparoscopic cholecystectomy, choledochotomy, and operations on the bile ducts. The second group involves operations of the upper abdomen, such as gastric resection, hepatectomy, pancreatic resection, lymphadenectomy, and other surgical procedures at close proximity to the hepatoduodenal ligament [1]. However, IBDI occur most frequently during either open or laparoscopic cholecystectomy [1]. The appropriate management of these injuries has been the subject of constant debate among experts. To date, several classification systems have been proposed, aiming to categorize these injuries [1]. The Bismuth classification [2], published in 1982, and the Strasberg classification [3] proposed almost a decade later, were anatomically oriented, classifying patients according to the location of the injury in the biliary tree (Figure 1). Since then, several attempts using the same methodological principles have been made, leading to the McMahon [4], the Bergman [5], the Neuhaus [6], and the Csendes [7] classifications.

However, the important impact of a concomitant vascular injury on the final outcome was not appropriately appreciated in any of these systems. Only recent systems, such as the Stewart-Way classification [8] published in 2007, the Hannover classification [9], and the ATOM (Anatomic, Time Of detection, Mechanism) classification [10], published by the European Association for Endoscopic Surgery (EAES) in 2013, have incorporated information regarding the possible presence of a concomitant vascular injury.

A state-of-the-art classification system should create groups of patients of similar prognosis but with distinct clinical characteristics. Specifically, for IBDI patients, prognosis relies additionally upon the complexity of the required treatment. Both the direct physiological consequences of the injury itself and the possible complications associated with the selected treatment determine the overall morbidity and mortality. The existing classification systems do not take into account that the management of IBDI is a dynamic field. The outcome of every certain treatment should be reckoned in the grading equation and influence, in a dynamic manner, the actual stage. For example, a bile leak that resolves spontaneously within 48 h renders the patients in a completely different prognostic environment than a bile leak that requires some kind of intervention. In addition, a classification system that is useful in everyday practice should dictate and guide appropriately the management of IBDI patients. The purpose of the present review paper is to propose a novel, simple, dynamic in nature classification system of IBDI by reviewing the relevant literature. However, our goal is not only to propose another classification system of IBDI, but to provide an evidence-based algorithm for the management of these patients.

## 2. Methods

### 2.1. Study Protocol

The present study was conducted in accordance with the Preferred Reporting Items for Systematic Reviews and Meta-Analyses (PRISMA) guidelines [11] and the Cochrane Handbook for Systematic Reviews of Interventions [12].

### 2.2. Eligibility Criteria

All human prospective or retrospective studies that reported relevant and retrievable data on IBDI were considered eligible. Exclusion criteria included the following: (1) non-human studies; (2) studies for which we were unable to gain full text access; (3) pediatric patients; (4) non-English language; (5) case reports; and (6) studies in the form of expert opinions, editorials, conference abstracts, and letters.

### 2.3. Literature Search

A systematic literature search was performed using the scholar databases Medline (PubMed), Scopus, and the Cochrane Library. The search included articles from the beginning of the databases to 20 March 2023; all studies published up to the last search date were included in the database screening. The following Boolean search algorithm was applied:

- [Iatrogenic] AND [Bile duct] AND [Injury]

In addition, the reference lists of eligible studies were screened manually.

### 2.4. Study Selection and Data Collection

After the removal of duplicate data, both titles and abstracts of the remaining studies were screened. This was followed by a full-text review. The literature screening, data extraction, and quality assessment were completed blindly and in duplicate by two independent researchers. In cases that a discrepancy could not be resolved, the opinion of a third senior researcher was considered. Quality and methodology evaluation included the Case Series Quality Appraisal Checklist for case-series studies.

### 2.5. Results

**Figure 2:**
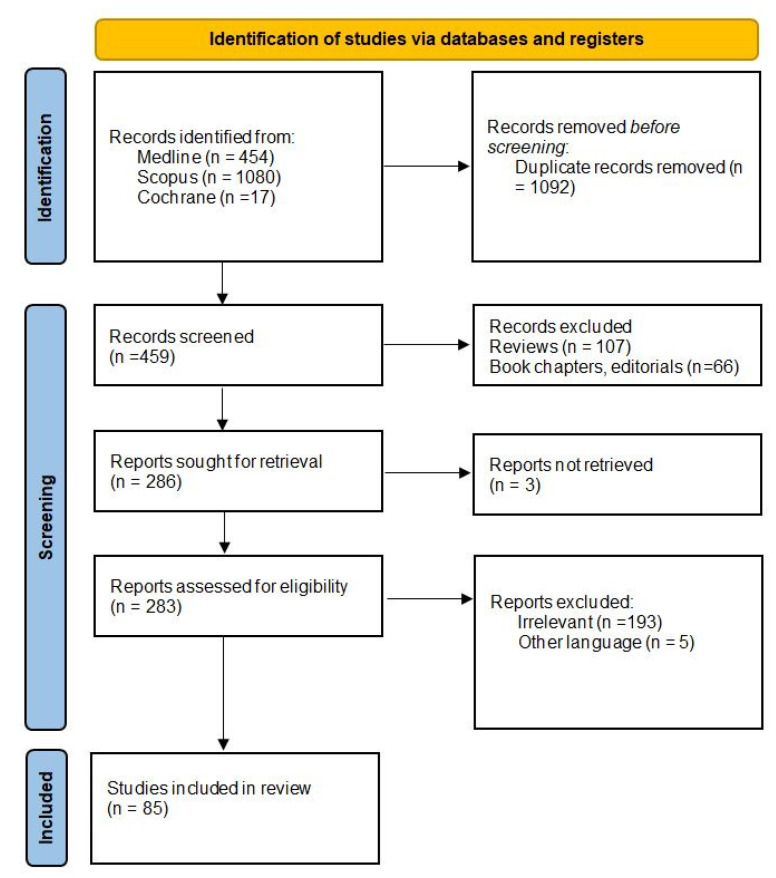
The flow chart of the study.

In total, 1551 records were identified from the databases. After the removal of the duplicated records, reviews, book chapters, and articles based on expert opinions, 286 reports were sought for retrieval. Finally, after the removal of those records not retrieved and the irrelevant and non-English language records, 85 articles relevant to IBDI were included in the present review (Figure 2).

## 3. Intraoperative Diagnosis

The diagnosis of IBDI can be made either intraoperatively, during the index operation, or during the postoperative period. The presence of an unexplained source of bile during either laparoscopic or open cholecystectomy should raise the level of suspicion for a possible IBDI, particularly partial or complete severance of the common hepatic duct or insufficient seal of the cystic duct [13]. Of note, this would not raise suspicion for other types of injuries including the occlusion of the common hepatic duct or a vascular injury. Several adjuncts have been proposed in order to establish a quite accurate intraoperative diagnosis.

Intraoperative cholangiography (IOC), although its routine use is not currently advised, may be of significant value in defining the anatomy of the biliary tree when an injury is highly suspected [13]. Along with indocyanine green fluorescence cholangiography (ICGC), conventional IOC may have a role in the setting of IBDI prevention as well [14]. Common indications of the two techniques in the injury prevention setting include the delineation of the relevant anatomy in high risk IBDI cases such as patients in whom the critical view of safety cannot be technically achieved or in patients with acute cholecystitis [14]. Currently, IOC represents the gold standard for assessing the anatomy of the biliary tree, as a part of an injury prevention strategy in challenging and complex cholecystectomy cases. However, there are several drawbacks associated with this approach that limit its widespread use, such as the increased operative time and the risk of even provoking an injury during IOC [14,15]. On the other hand, ICGC aims to address the limitations of conventional IOC and to provide a safe, real–time, and non-invasive visualization of the extrahepatic biliary tree anatomy during laparoscopic cholecystectomy [16,17]. A recent meta-analysis showed at least similar, if not increased, cystic duct, common bile duct, cystic duct-common bile duct junction, and common hepatic duct visualization rates by ICGC compared to IOC [16].

Intraoperatively, the establishment of an accurate diagnosis of an IBDI is the prerequisite for a successful repair. However, in the case of insufficient experience in hepatopancreatobiliary (HPB) surgery, it is highly recommended to place a drain and refer the patient to a specialized center [18]. Converting to open surgery just to confirm the diagnosis and perform injury staging is not recommended. A recent systematic review and meta-analysis of 32 studies showed that the rate of repair failure was significantly higher for early versus delayed repair, and was lower for early versus delayed referral to an expert center [19]. In addition, non-expert immediate repairs were associated with the worst outcomes [19,20]. When sufficient HPB experience is available, there are conflicting literature reports in regards to the optimal timing of the repair. There are studies suggesting a benefit from early repair [21,22,23], while other studies did not demonstrate a difference between on table, early (within 72 h), or postoperative repair (within 1 week), provided that the repair is undertaken by specialized HPB surgeons [19,24,25].

## 4. Clinical Consequences of Bile Duct Injuries

If an IBDI is not diagnosed during the index operation, then the consequences of the injury become obvious during the postoperative period, mainly as a bile leak orobstructive jaundice [26]. In general, IBDI include a wide variety of injuries ranging from bile leaks to bile duct occlusion and vascular injuries. There might be injuries to adjacent organs as well, i.e., the duodenum and colon, which, although not directly representing bile duct injuries, could have implications on and confound immediate and future diagnosis and therapy.

Regarding bile leaks, if a drain was left in place, then a biliary fistula will be established. In cases with no drain in place, bile leaking through the site of the injury will accumulate within the abdominal cavity, resulting either in a contained perihepatic collection, i.e., biloma, or in a progressive, diffuse, biliary peritonitis. Attempting to assess the severity of bile leaks, Sandha et al. proposed a classification system for bile leaks [27]. Bile leaks were classified as low grade and high grade, by the use of endoscopic retrograde cholangiopancreatography (ERCP). Low grade leaks were defined as those leaks that were identified fluoroscopically only after the opacification of the intrahepatic radicles during ERCP. It is hypothesized that a low grade leak indicates the presence of a minor injury, which only becomes evident after a notable increase of the intraductal pressure. On the other hand, a high grade leak is observed well before the opacification of the intrahepatic bile ducts, suggesting that the defect is large enough to become evident even after a slight increase of the intraductal pressure [27]. A biliary sphincterotomy alone could effectively decrease the intraductal pressure and guide the bile flow towards the papilla, resulting in the closure of a low grade leak. However, sphincterotomy alone may be insufficient to allow the healing of a defect associated with a high grade leak, and thus, stent placement, with or without sphincterotomy, has been proposed as the preferred initial treatment for high grade leaks [27].

On the other hand, IBDI causing obstruction of the extrahepatic biliary tree will result in the clinical syndrome of obstructive jaundice, often complicated with acute cholangitis due to bacterial overgrowth. Recurrent episodes of acute cholangitis can result in biliary cirrhosis with the subsequent development of portal hypertension and liver failure [28]. Delayed diagnosis of the stricturing type of injuries is not uncommon, as symptoms may be rather atypical, increasing substantially the morbidity and the mortality rates even after surgical treatment [29].

## 5. Postoperative Imaging Investigation

In the postoperative setting, the imaging evaluation of patients with suspected IBDI is particularly important. The role of imaging is to establish the diagnosis and determine the type and the extent of the injury.

Ultrasonography (US) is a readily available, low-cost modality, and usually represents the first diagnostic modality performed. Despite its limitations, US can effectively detect fluid collections, bile duct dilations, and, with the use of Doppler, associated vascular lesions [30].

Abdominal triple phase contrast computed tomography (CT) scan is considered more accurate than the US in the workup of IBDI patients. CT can identify the possible presence of fluid collections, ascites, biliary obstruction with proximal dilation up to the level of intrahepatic bile ducts, and hepatic atrophy associated with a long-standing stricture or signs of secondary biliary cirrhosis [31,32]. However, the main advantage of CT is that it can provide an accurate assessment of the arterial anatomy and identify concomitant vascular injuries, such as injuries to the right hepatic artery [33]. CT has superior sensitivity over the US, especially for the detection of small fluid collections and associated vascular complications [33,34]. Both US and CT have an additional, well-defined role in the treatment algorithm by permitting the localization of collections and performing percutaneous drainage procedures [34]. However, because of their similar densities, neither of them can effectively distinguish the nature of the postoperative fluid collections, i.e., whether the collection consists of bile, blood, pus, or serous fluid. In addition, these modalities cannot determine the precise source of a bile leak [35].

Hepatobiliary scintigraphy (HS) seems to be more sensitive and specific than US or CT in detecting bile leaks, and in identifying the relationship between a leak and any fluid collection [36]. HS can demonstrate the presence of an active bile leak, but usually without identifying the primary leak site due to its limited spatial resolution [36,37]. The sensitivity of HS is rather poor in patients with deranged liver function and large bile duct defects [36].

ERCP and percutaneous transhepatic cholangiography (PTC) can be especially useful in identifying a continuing bile leak and providing an accurate anatomical diagnosis, while they have a quite decisive role in the treatment algorithm [38]. According to each individual case scenario, the decompression of the biliary tree, the dilatation of a stricturing injury or the insertion of biliary stent can be effectively achieved via ERCP [38,39]. Success rates increase if the duct injury is extrahepatic, <5 mm in diameter, and it is not associated with the presence of an abscess [39]. Whenever ERCP fails, PTC is the valid alternative. PTC can prove quite challenging technically, in the setting of an active bile leak due to the lack of intrahepatic bile duct dilation. The combination of the two techniques in the form of the percutaneous-endoscopic rendezvous procedure has been reported as safe in patients with complex IBDI requiring the reestablishment of bile duct continuity, either as a definitive treatment or as a bridge to elective surgery, with a long-term success rate of up to 55% [40]. The drawback of ERCP and PTC is that they are invasive techniques, and thus, they are associated with a non-negligible risk of complications [39,40]. Severe acute pancreatitis, bleeding, perforation, and cholangitis, especially after PTC, represent the most common complications [38,41].

The limitations of these invasive techniques, i.e., ERCP and PTC, as diagnostic modalities are compensated for, almost in total, by magnetic resonance cholangiopancreatography (MRCP) [35,42]. MRCP currently represents the “gold standard” for the evaluation of the biliary tree after IBDI. It is non-invasive and, unlike ERCP and PTC, provides accurate anatomical visualization of the biliary tree both proximal and distal to the level of injury [42]. Contrast-enhanced MRCP with intravenous hepatocyte-selective contrast agent with biliary excretion allows for the detection and localization of bile leaks with a reported accuracy of up to 100% [42]. Several studies have confirmed that contrast-enhanced MRCP—using 3D and 2D T1-weighted images after the intravenous injection of hepato specific contrast agents—improves the accuracy of bile duct anatomy visualization and bile leak detection rates [43,44]. In a study with 99 patients, contrast-enhanced MRCP proved significantly more accurate (84% vs. 58%) and sensitive (79% vs. 59%) in bile leak localization than conventional MRCP [45]. When identifying the source of bile is the actual challenge, the optimal timing for hepatobiliary phase image acquisitions with contrast-enhanced MRCP appears to range between 60 and 90 min [46].

## 6. Treatment Strategy

In general, the treatment of IBDI requires a multimodality approach. Several specialties need to work, as a solid team, for the optimal outcome. In regards to treatment, the available options are as follows: 1. Conservative management with or without the administration of antibiotics; 2. Sepsis control strategies, i.e., percutanous radiologic drainage of intra-abdominal bilious or septic collections or surgical drainage; 3. ERCP or PTC; 4. Surgical repair, such as performing an end to end ductal anastomosis or a Roux en Y hepaticojejunostomy; and 5. Hepatic resection and/or transplantation.

A step-up approach—from purely conservative treatment to interventional/endoscopic techniques, and ultimately, to surgical repair—usually summarizes the treatment decision-making process for IBDI diagnosed in the postoperative setting [18,21]. For intraoperatively diagnosed injuries, the “drain now and fix later” strategy seems appropriate when the necessary expertise in advanced HPB procedures is lacking from the surgical team [47]. On the other hand, a definite repair approach upfront, during the index operation, could be attempted, provided that an experienced HPB surgeon is available on site [18]. The repair technique followed is influenced by the type of injury [18].

The presence of a concomitant vascular injury should be taken into account, and influence the decision-making process. In general, arterial and portal vein reconstructions are complex procedures. A thorough workup with contrast-enhanced CT is commonly required in order to accurately assess the extent of the injury. The right hepatic artery is the most commonly injured vessel, with the incidence in the literature ranging 10–47% among patients with IBDI [48]. In general, the hepatic blood supply comes predominantly from the portal venous system, and thus, the ligation of the hepatic artery is usually well tolerated [49]. In addition, intrahepatic and prehepatic vascular collaterals try to preserve the blood flow to the affected liver parenchyma and prevent hepatic necrosis in case of an arterial injury [49]. However, factors such as the hemodynamic stability of the patient, the location of the injury, and the presence of comorbidities can influence the ischemic effect on the liver [49,50,51]. The concomitant biliary obstruction, on the background of an IBDI, may further predispose to the development of hepatic necrosis [49,50,51]. As soon as a liver parenchymal necrosis develops, the subsequent bacterial contamination can lead to abscess formation and sepsis [51]. Patients submitted to any form of preoperative biliary drainage procedure are at high risk of developing these septic complications [49,52,53]. In general, these complex, combined injuries require an extensive workup in the direction of the precise anatomical classification of the injury.

## 7. The BILE Classification

We propose a five (5) stage (A, B, C, D and E) classification system for IBDI (BILE Classification) (Table 1). Each stage is correlated with the recommended and most appropriate treatment. Although the proposed classification scheme is clinically oriented, the anatomical correspondence of each IBDI stage using the Strasberg classification has been incorporated (Figure 1) (Table 1) [3].

### 7.1. Grade A

These injuries are usually bile leaks that can be treated conservatively, at least initially. This conservative management includes inpatient observation with the administration of parenteral broad-spectrum antibiotics if signs of infection are present. The cessation of oral feeding is not usually required.

Bile of unknown origin detected during the index operation should raise the level of suspicion for a possible IBDI. The dilemma in this setting is whether to proceed to an immediate repair or to defer the repair to a later stage after the proper drainage of the subhepatic area. The immediate repair option requires an accurate assessment of the type of injury using diagnostic adjuncts such as IOC, and appropriate experience in any type of often complex repairs that might be required [19,20,47]. However, as laparoscopic cholecystectomy is one of the most common operations performed, surgeons who lack the required expertise in advanced HPB operations usually face the problem of dealing with an IBDI [47]. Converting to open surgery just to confirm and classify an IBDI is not recommended. Although there were reports of favorable results when immediate repair was attempted, a recent meta-analysis showed that early referral and delayed repair appear to confer the more favorable outcomes [19]. From the viewpoint of the authors, the “drain now, fix later” approach, as a recommendation, can be generalized more readily than the immediate repair approach.

In Grade A IBDI diagnosed in the postoperative setting, the usual clinical scenario involves a patient after laparoscopic or open cholecystectomy with a bile leak from a drain left in situ during the index operation. A thorough imaging investigation in the direction of an accurate assessment of the injury type and possible presence of other conditions requiring intervention such as contained collections is warranted. In general, a trial of observation and non-operative management is advisable during the first hours because the timing of a possible endoscopic treatment, relatively to the discovery of the bile leak, does not seem to affect the overall success rates [18,54,55]. The concept is to allow time for the leak to resolve spontaneously. Antimicrobial therapy might not be necessary. However, antibiotics are required if signs of infection are present [56]. In this case, parenteral broad-spectrum antibiotics should be started and subsequently appropriately adapted to bile and blood cultures. The most commonly used antibiotics are piperacillin/tazobactam, imipenem/cilastatin, meropenem, ertapenem, or aztreonam associated with amikacin in cases of associated shock [56]. Fluconazole should be added in the antibiotic regimen in cases of fragility or delayed diagnosis [56].

From the anatomical viewpoint, these injuries correspond to Strasberg A, C, and D injuries. These injuries refer to bile leaks originating from the cystic duct or the liver parenchymal surface where the gallbladder is attached, from a transected aberrant right hepatic duct that is not communicating with the common bile duct (CBD) or from a lateral laceration, without however, loss of continuity of the CBD. Often, these lateral lacerations of the CBD might require additional interventions, but as soon as the assessment of the bile duct circumference, affected by the injury, cannot be accurately assessed during the imaging workup, an initial short trial of conservative treatment appears justified [54,55].

### 7.2. Grade B

These injuries represent as anatomically equivalent to Grade A injuries in patients that require some kind of intervention in the direction of sepsis control [57,58]. Available options include the percutaneous drainage, usually under CT guidance, of a contained intra-abdominal collection (biloma), and the surgical (open or laparoscopic) lavage and drainage of the peritoneal cavity on the background of diffuse biliary peritonitis. The goal is to control sepsis and get the patient into a stable condition for any further interventions [57,58]. Several days might be required for the resolution of the systemic inflammatory response [57,58].

A bile leaking injury after cholecystectomy with no drain in situ can lead to the formation of a biloma or to a progressive diffuse biliary peritonitis. Both of these conditions require some sort of intervention. An inadequately drained biliary fistula causing systematic septic effects and associated with localized intrabdominal collections warrants similar management. Unlike Grade A injuries, antibiotics are usually required in the setting of Grade B injuries because a postoperative septic patient profile is clinically evident and confirmed by the laboratory test results and the appropriate imaging studies [57,58,59,60]. The need for an intervention with the intent to control sepsis and not to address the injury itself in a definite manner is the required condition for assigning patients into this category.

### 7.3. Grade C

These are the bile duct injuries qualified for endoscopic or percutaneous treatment, usually via ERCP. Grade A or B injuries that fail to improve, usually after a short trial of conservative and/or interventional/laparoscopic drainage management, respectively, should be considered as Grade C injuries. Several endoscopic treatments such as biliary stenting, biliary sphincterotomy, and nasobiliary drainage have proved highly effective in treating bile leaks [61]. The endoscopic management is indicated when there is at least partially documented continuity of the BDI (at the MRCP). A complete common bile duct transection represents a traditional contraindication for the endoscopic treatment [61].

In general, when addressing a bile leak, the main goal of endoscopic therapy is to reduce the transpapillary pressure gradient and direct the bile to flow preferentially towards the papilla rather than the site of the leak. The most common approach to achieve this is to place a plastic stent within the bile duct through the ampulla of Vater. Naso-biliary drainage has also been tested in this direction, showing similar efficacy to plastic stents but with the drawback of limited patient compliance [62]. The role of sphincterotomy alone in the management of these patients is not quite clear. Certainly one major ERCP related complication, i.e., perforations emanate for this certain part of the procedure, and the utilization of sphincterotomies under the proper indications is a wise approach [63]. In practice, the most frequently followed approach is the combination of endoscopic sphincterotomy along with the placement of a plastic biliary stent; this approach results in the closure of the leak in >90% of patients [62,63,64]. The outcomes of endoscopic treatments are superior for leaks of low output, but high output leaks can be effectively managed as well [27].

In regards to stent characteristics, short transpapillary plastic stents which reduce the intraductal pressures have been shown to be equally effective as longer ones which additionally bridge the leak site [63,65]. Biliary stents are usually removed after 4 to 6 weeks, a time period which allows the complete healing of the leak in the majority of cases [66]. The impact of the diameter of the stent on the healing rates has been debated. The majority of the relevant studies did not reveal any benefits on the outcomes of the use of larger diameter stents, i.e., 10 Fr vs. 7 Fr [67,68]. However, large diameter stents (10 Fr) are preferred by endoscopists in order to avoid early clogging [69,70]. In addition, after sphincterotomy, the use of a large stent (10 Fr) could reduce the risk of stent migration [63].

For refractory bile leaks, no clear strategy has been proposed. Currently, two approaches have been used in these challenging cases, i.e., the temporary placement of a fully covered self-expandable metal stent, and the use of more than one plastic stents [71,72]. The placement of multiple large-caliber plastic stents—ideally ≥3, summing to at least 20 Fr—or a fully covered self-expandable metal stent are approaches with expected closure rates of >90% [71,72,73]. All types of treatment with multiple plastic stents for refractory biliary leaks that are not successful should cross over to a fully covered self-expandable metal stent that should stay in place for no longer than 3 or 4 weeks [74]. In general, the overall success rates and the adverse effects after ERCP for the treatment of bile leaks do not seem to be dependent on the timing of the endoscopic procedure relative to the discovery of the bile leak [54,55]. This suggests that ERCP in these patients can be usually performed in an elective, rather than an urgent, manner [54,55].

While the endoscopic techniques have a role in treating bile leaks, usually as a second-line option in the step-up approach strategy, they should be considered the first-line treatment when an iatrogenic biliary stricture is the case [54,55]. These injuries can be diagnosed either in the immediate postoperative period or relatively late, even years after the index operation. In general, strictures recognized early respond satisfactorily to endoscopic maneuvers, unlike strictures occurring late after IBDI [75,76]. The European Society of Gastrointestinal Endoscopy (ESGE) recommends the temporary insertion of multiple plastic stents or of a fully covered SEMS for the treatment of benign biliary strictures [74]. A recent meta-analysis of eight randomized control trials comparing covered self-expanding metal stents with multiple plastic stents for benign biliary strictures did not reveal any differences in regards to stricture resolution, recurrence of stricture, stent migration, and moderate-severe adverse events between the two approaches [77]. However, self-expanding metal stents required fewer sessions of ERCP for stricture resolution than the multiple stenting approach [77].

In regards to the plastic stents strategy, the current approach consists of inserting an increasing number of plastic stents every 3–4 months. The treatment is terminated after the complete morphologic disappearance of the stricture, or after fixed 12-month stenting duration [74]. The simultaneous placement of multiple plastic stents for benign strictures of the CBD is technically feasible in >90% of the cases [74,76]. For the treatment with fully covered self-expanding metal stents, the ESGE recommends insertion of an 8–10-mm diameter stent for a dwell stenting period of 6 months [74]. In general, endoscopy provides the highest long-term biliary patency rate in 90% of postoperative biliary strictures, but the non-negligible recurrence rates after the stent removal dictate a close follow up scheme [76,78,79].

When the endoscopic approach is unsuccessful or not feasible, the percutaneous approach, in the form of percutaneous transhepatic cholangiography (PTC), is the alternative [80]. From the technical viewpoint, PTC is often non-feasible in the absence of dilated bile ducts; however, it is especially helpful when a complete obstruction of the CBD precludes the endoscopic access, via ERCP [80,81]. In such cases, PTC is used for draining the biliary tree before the definite intervention, especially when treating recurrent episodes of acute cholangitis is the actual challenge. Furthermore, PTC is often the only way to access the biliary tree after a failed surgical repair, i.e., Roux en Y hepaticojejunostomy [81].

Recently, the role of endoscopic ultrasound-guided biliary drainage (EUS-BD) has gained considerable attention as an approach that can provide relief in malignant and benign biliary obstruction for patients when ERCP fails or is not feasible [82]. Endoscopic ultrasound-guided biliary drainage can be classified into two major groups: the transpapillary approach (rendezvous retrograde and antegrade stent insertion) and the transmural approach (choledochoduodenostomy and hepaticogastrostomy) [82]. The former involves the transgastric or transduodenal creation of a temporary pathway in which a guidewire is advanced through the biliary tree across the ampulla and into the duodenum in order to allow conventional ERCP, while the latter involves a transgastric puncture into the left intrahepatic biliary system with passage of a guidewire into the duodenum [82]. The track is dilated to allow passage of a stent into the CBD and across the papilla without creating an anastomosis at the puncture site [82]. A meta-analysis comparing PTC with EUS-BD, in the setting of failed ERCP, concluded that there was no difference in technical success rates between the two procedures, but EUS-BD was associated with better clinical success, fewer post-procedure adverse events, and lower rates of re-interventions [83]. Currently, EUS-BD is increasingly used for benign disease as an alternative to percutaneous approaches in centers with high expertise [82,83].

A symptomatic, Strasberg B injury falls into this category as well. A Strasberg B injury involves the occlusion of a right posterior sectorial duct without bile leak. Mild pain and elevation of liver function tests can be present, but usually without significant clinical impairment [29]. As the clinical signs are often insidious, unless suspected otherwise based on the intraoperative findings, such injuries can be easily missed. Luckily, only a minority of this type of injury will require additional interventions. A percutaneous drainage of the occluded part of the biliary tree, manifested with relapsing episodes of acute cholangitis or liver abscess formation, represent a valid treatment option. From the anatomical viewpoint, Grade C injuries include all grade A and B injuries where the initial adopted conservative treatment strategy failed, and the Strasberg E1–E5 stricturing injuries.

### 7.4. Grade D

Grade D are the injuries that require surgical repair. Surgical repair is mainly summarized in an end to end ductal anastomosis, usually over a T-tube, or in the form of a Roux en Y hepaticojejunostomy [84]. Direct repair with suturing of the site of the injury with or without the use of a T tube for intraoperatively diagnosed injuries has been also proposed [85]. The early surgical repair, usually within 48 h from the diagnosis of this kind of injury, appears to be associated with the most optimal results [86]. The rationale is that the prompt intervention, before the establishment of sepsis, leads to decreased morbidity rates, reduced costs, and rates of hospital readmissions [86,87]. The threshold of 72 h has been considered the turning point where the healing process is initiated, complicating the surgical repair [85].

However, the authors discourage this approach. Deciding to proceed in diagnosing and repairing a suspected IBDI during the index operation should be regarded as a one-way road. Apart from the fact that the diagnostic evaluation cannot be entirely accurate, unexpected findings requiring complex and time-consuming reconstructions might be encountered. An inadequately prepared surgical team with limited expertise in advanced HPB procedures might do more harm than good, compromising the success of the whole treatment strategy. Late repairs should be considered whenever septic complications need to be first addressed prior to proceeding to the surgical repair. In these complicated settings, the late repair is associated with the most optimal results compared to the early and immediate repair, i.e., mortality and morbidity rates of late, early, and immediate repair of 0.8% vs. 2.8% vs. 2.2%, and 14.3% vs. 39.2% vs. 28.7%, respectively [88].

Apart from the time factor, the experience of the index surgeon in HPB operations has been highlighted as an important determinant of the repair success [85]. Referral to a specialized setting has been suggested as a means of bypassing the handicap of limited experience in the field of HPB surgery [3,20,89,90].

In regards to the repair technique, a repair with an end to end ductal anastomosis, with or without the placement of a T-tube is an option with success rates that range significantly in the relevant literature [90]. However, a tension-free bilioenteric anastomosis usually in the form of a Roux en Y hepaticojejunostomy with good mucosal apposition and vascularized ducts is the mainstay of treatment [91]. Especially when significant tissue loss and a concomitant ischemic injury are present, the Roux en Y hepaticojejunostomy is the sole recommended approach. The incidence of repair failure, mainly in the form of anastomotic strictures, varies significant in the literature (between 4.1% and 69%) [90,91]. However, the majority of studies report an incidence of 10–20%, and a median time to stricture formation of 11–30 months [90,91]. The risk factors for the failure of the repair include the associated vascular injury, the anatomical location of the injury as defined in the Strasberg classification, the presence of sepsis, and the postoperative bile leakage [92].

The reconstruction using a hepaticoduodenostomy has been also proposed [93]. The shorter operative time, the decreased bleeding and the lower incidence of adhesive intestinal obstruction represent some of the theoretical advantages of the approach compared to Roux en Y hepaticojejunostomy [94]. However, surgical technical parameters limit the feasibility of this certain approach [95].

From the anatomical viewpoint, Strasberg E category injuries (E1–E5)—where the clinical scenario involves a complete transection, with loss of continuity of the CBD or of the hepatic duct, even up to the level of the left and right bile duct confluence—are the injuries that directly fall into this category. The status of the proximal stump, i.e., non-clipped or clipped will influence the clinical presentation, i.e., bile leak or progressive obstructive jaundice, respectively. In these injuries, the endoscopic treatment approaches are not feasible by definition, though there are reports of successful combined endoscopic-radiological approaches when the two non-clipped stumps are in close proximity [95,96]. However, the employment of such attempts requires high expertise, and generalizations regarding the indications could be risky. In addition, lateral injuries (Strasberg D) of the extrahepatic biliary tree might require surgical treatment as well. Although endoscopy is usually the first line treatment option for this kind of injury, recurrences are relatively common, rendering patients ultimately eligible for a Roux en Y hepaticojejunostomy [90,91].

### 7.5. Grade E

Grade E injuries are those requiring liver resection or liver transplantation. They usually involve patients with delayed diagnosis of a bile duct injury leading to permanent impairment of liver function, or patients for whom several treatment options have failed and bile duct injury patients with concomitant vascular injuries.

In regards to concomitant vascular injuries, in up to 25% of bile duct injury patients there is a concomitant right hepatic artery injury [97]. As the blood supply to the liver is carried out mainly by the portal vein, arterial injuries are usually well tolerated [91]. However, the effect that a concomitant vascular injury might exert on the final outcome is more or less unpredictable. A cumulative 10% actual incidence of clinically significant liver ischemia should be anticipated in cases of combined biliary and vascular injuries [98]. These injuries can be diagnosed intraoperatively or during the postoperative period. Given the most often favorable clinical course of these injuries, the high level of technical expertise required for attempting a repair and the low rates of early diagnosis, the immediate repair upon recognition during the index operation is not advocated for even in tertiary centers [98,99]. A “wait and see” strategy in order to avoid complex reconstructions of obscure necessity and to obtain an accurate imaging assessment appears justified [97,98]. However, these injuries can, indeed, increase morbidity and mortality rates. Hepatic atrophy and abscess formation and increased risk of leakage or stricture of a bilioenteric anastomosis represent the possible consequences of ischemia [53,99]. In general, the management of postoperatively diagnosed vascular injuries depends on the evidence and extent of the liver injury such as ischemia, necrosis, or atrophy. Surgical management should be delayed for several weeks and even months to allow for an accurate imaging workup and targeted interventions.

Hepatic resections of affected liver segments/lobe might be required. In general, only a small minority of IBDI will ultimately require a hepatectomy. A liver resection could be considered when several treatment approaches have failed and resulted in a non-functional liver segment/lobe which usually proves to be the cause of severe morbidity [100]. This mainly refers to patients with high vasculo-biliary injuries, many years after the injury presenting with liver atrophy and recurrent episodes of cholangitis and sepsis which are not amenable to other interventions. As hepatectomy on the background of IBDI is associated with substantial morbidity, careful patient selection is particularly important [100]. On the other hand, when the deterioration of liver function becomes irreversible, as assessed and confirmed by relevant liver function scoring systems such as the Child Pugh score or the model for end-stage liver disease (MELD) score, then the liver transplantation appears as the last resort [101]. Secondary biliary cirrhosis associated with liver failure in patients with combined biliary and vascular injuries is one of the limited indications for liver transplantation in the setting of IBDI [101].

## 8. Discussion

An ideal classification system should classify patients in a simple, yet informative way of increased prognostic validity. Certainly, when the development of IBDI preventive strategies or analyzing epidemiological or comparative data is the actual goal, an analytical and extensive classification system able to discriminate every single different IBDI case scenario is the proper tool. However, everyday clinical practice screams out for practicality and simplicity. Extensive classification themes such as the ATOM system [9], though accurate and precise, are too complex and, obviously, time consuming. Within this framework, the penetration of such themes in the involved settings can prove quite problematic.

In the present review, we propose a simple, novel classification system of IBDI of increased clinical utility by systematically reviewing the relevant literature. BILE classification differs from previously published classification schemes because it additionally provides an action map that can appropriately guide the treatment plan in this challenging setting. Unlike previous attempts, the present classification and treatment algorithm focuses on the clinical consequences of IBDI. The dynamic nature of the therapeutic strategy has been taken into account and has been incorporated, in an evidence-based manner, in the decision-making process. However, a notable limitation is that our proposed classification has not been properly validated in a prospective patient cohort. As by definition, IBDI is a clinical scenario with vast variations and notable diversity, possible reliability and prognostic implications issues of the proposed classification should be kept in mind. In addition, there are not always high-quality literature data available to draw accurate conclusions and determine the appropriate, evidence-based strategic planning.

## 9. Conclusions

The management of patients with IBDI is a challenging field with often dismal medico legal projections. An accurate action map—starting from the point of clinical suspicion of the occurrence of such injury and ending at the time of the definite treatment—is more than necessary. Attempts to classify IBDI have been made repeatedly and the final results were either analytical and extensive but not useful in everyday clinical practice systems, or simple and user friendly but with limited clinical correspondence approaches. BILE classification correlates, in a strict and reproducible way, each IBDI stage with the most appropriate treatment in a balanced, simple, yet informative classification scheme. The validation of our proposed classification in a prospective patient cohort could further prove its clinical utility.

## Figures and Tables

**Table 1:** The BILE Classification of IBDI.

	Clinical Presentation	Anatomical Description and Site of the Injury (Strasberg Classification)	Treatment
**Grade A**	Biliary fistula (drain in situ)	A, C, D	Conservative management
**Grade B**	Biloma Biliary Peritonitis	A, C, D	Percutaneous drainage or surgical lavage and drainage
**Grade C**	Biliary fistula not responding to conservative management CBD Stricture with obstructive jaundice or recurrent episodes of acute cholangitis	A, B (Symptomatic), C, D, E1–E2	ERCP/PTC
**Grade D**	CBD stricture not sufficiently addressed by ERCP/PTC Complete transection of the CBD presenting as bile leak or obstructive jaundice	D, E1–E5	End to end ductal anastomosis Roux en Y Hepaticojejunostomy (Gold Standard)
**Grade E**	Liver atrophy, abscess due to concomitant vascular injury or delayed bile duct injury diagnosis Liver failure	-	Liver Resection Liver Transplantation

CBD: Common bile duct; ERCP: Endoscopic retrograde cholangiopancreatography; PTC: Percutnaeous transhepatic cholangiography.

## Data Availability

The datasets used and/or analyzed during the current study are available from the corresponding author on reasonable request.
